# The Efficacy of an Immunoisolating Membrane System for Islet Xenotransplantation in Minipigs

**DOI:** 10.1371/journal.pone.0070150

**Published:** 2013-08-01

**Authors:** Tova Neufeld, Barbara Ludwig, Uriel Barkai, Gordon C. Weir, Clark K. Colton, Yoav Evron, Maria Balyura, Karina Yavriyants, Baruch Zimermann, Dmitri Azarov, Shiri Maimon, Noa Shabtay, Tania Rozenshtein, Dana Lorber, Anja Steffen, Udi Willenz, Konstantine Bloch, Pnina Vardi, Ran Taube, Paul de Vos, Eli C. Lewis, Stefan R. Bornstein, Avi Rotem

**Affiliations:** 1 Technology Department, Beta-O2 Technologies, Rosh HaAyin, Israel; 2 Department of Medicine III, University Hospital Carl Gustav Carus, Dresden, Germany; 3 Islet Department, Beta-O2 Technologies, Rosh HaAyin, Israel; 4 Section of Islet Trasnplantation and Cell Biology, Joslin Diabetes Center, Boston, Massachusetts, United States of America; 5 Department of Chemical Engineering, Massachusetts Institute of Technology, Cambridge, Massachusetts, United States of America; 6 Veterinary Department, Lahav Research Institute, Lahav, Israel; 7 Diabetes and Obesity Research Laboratory, Faculty of Medicine - Tel Aviv University (Beilinson Campus), Petah Tikva, Israel; 8 Department of Microbiology Immunology and Genetics, Ben-Gurion University of the Negev, Beer-Sheva, Israel; 9 Department of Pathology and Laboratory Medicine, University Medical Center Groningen, Groningen, The Netherlands; 10 Department of Clinical Biochemistry and Pharmacology, Ben-Gurion University of the Negev, Beer-Sheva, Israel; University of Bremen, Germany

## Abstract

Developing a device that protects xenogeneic islets to allow treatment and potentially cure of diabetes in large mammals has been a major challenge in the past decade. Using xenogeneic islets for transplantation is required in light of donor shortage and the large number of diabetic patients that qualify for islet transplantation. Until now, however, host immunoreactivity against the xenogeneic graft has been a major drawback for the use of porcine islets. Our study demonstrates the applicability of a novel immunoprotective membrane that allows successful xenotransplantation of rat islets in diabetic minipigs without immunosuppressive therapy. Rat pancreatic islets were encapsulated in highly purified alginate and integrated into a plastic macrochamber covered by a poly-membrane for subcutaneous transplantation. Diabetic Sinclair pigs were transplanted and followed for up to 90 days. We demonstrated a persistent graft function and restoration of normoglycemia without the need for immunosuppressive therapy. This concept could potentially offer an attractive strategy for a more widespread islet replacement therapy that would restore endogenous insulin secretion in diabetic patients without the need for immunosuppressive drugs and may even open up an avenue for safe utilization of xenogeneic islet donors.

## Introduction

Islet transplantation for patients with type 1 diabetes is still an infrequently applied therapeutic approach performed only in highly specialized medical centers. Long-term clinical outcomes of this approach have improved gradually over the past decade [1]. Islet transplantation is superior to intensive insulin therapy in selected patient groups [2] and can be almost as successful as transplantation of a whole pancreas, due to optimized islet isolation/culture procedures and innovative immune strategies [3]. However, the chronic need for immunosuppressive therapy following islet transplantation and the persistent shortage of high-quality donor organs is currently restricting this therapeutic approach to a group of high-risk patients who have exhausted conservative treatment options. Indeed, only patients with unstable metabolic control, repeated severe hypoglycemia that is often associated with hypoglycemic unawareness, or those with rapidly progressive diabetes-associated complications are eligible for islet transplantation in most centers [4]. Furthermore, a thorough risk-benefit analysis is required to justify immunosuppressive therapy in patients suffering from a generally non-acute life-threatening disease [5].

When islets are immunoisolated in immunoprotective membranes, chronic use of immunosuppressive therapy is not required (at least in theory), as enveloping donor islets in these membranes protects them against the deleterious effects of the host immune system, thereby making the use of xenogeneic grafts feasible. However, the number of reports on transplantation of xenogeneic islets in large mammals is scarce and the reported success rates are highly variable [6]–[8]. It has been postulated that the cause for this limited success is a far stronger immune response provoked by transplanted xenogeneic islets than allogeneic islets, a response against which membranes alone cannot protect [9], [10]. The assumed mechanism involves leaking of the highly immunoreactive epitopes on xenogeneic islets, such as galactosyl residues, and their reaction with naturally occurring (anti-Gal) and non-Gal IgM antibodies, which in turn, activates the classical complement pathway and induces neutrophil infiltration near the graft [11]. This IgM-mediated humoral reaction against the enveloped xenogeneic islets can also induce the typical delayed-type hypersensitivity response associated with xenografts and does not necessarily need cell-to-cell contact such as in allogeneic responses. The current generation of membranes is considered incapable of protecting a graft against these types of responses [12].

Another major challenge in the development of a successful bioartificial pancreas is designing a device that can carry a large enough volume of islets to achieve normoglycemia, yet would be small enough to be transplanted without undesired side effects in large animals and humans. Oxygen supply is also a crucial factor for the success of the device. Some success has been reported in a xenogeneic large animal transplantation model using porcine islets transplanted into a well-perfused site [6]. However, the dose of islets used in a similar experiment with macroencapsulated islets was very high [13], making the size of a corresponding device for humans impractical for clinical use.

To overcome the aforementioned limitations of oxygen supply and of immunoisolating membranes for xenografting, we designed a novel device with a 3-component gas chamber and a membrane that is impermeable to complexes required for the activation of the xenogeneic rejection processes. To this end, we applied a macroencapsulation approach in which we used a multilayer immunoprotective membrane of alginates and a polytetrafluoroethylene (PTFE) membrane. We studied the retention and permeability of the membrane for immunoglobulins while simultaneously allowing for sufficient supply of oxygen for optimal function of the islets. In a previous study, we have demonstrated the functional potency and immunoprotective characteristics of similar devices using allogeneic transplantation in a rodent model system and in a large animal model [14], [15]. In this paper, we describe an improved device (it includes increased islet biomass, a better gas ventilation system, and a modified immune barrier) and its efficacy in a large animal diabetes model (streptozotocin [STZ]-induced diabetic minipigs) by evaluating long-term (up to 90 days) function of the islets and the capacity of the system to protect the xenograft from immunologic attacks without the use of immunosuppressants.

## Materials and Methods

### Ethics Statement

All animal experiments were performed in strict accordance with guidelines established by the Israeli Institutional Animal Care and Use Committee (IACUC). The study was approved by the Israeli IACUC (permit number IL-11-10-173, issued by the chairman of the Israeli IACUC to Beta-O_2_ technologies). All efforts were made to minimize animal suffering.

### Implantable Macrochamber

**Figure 1:**
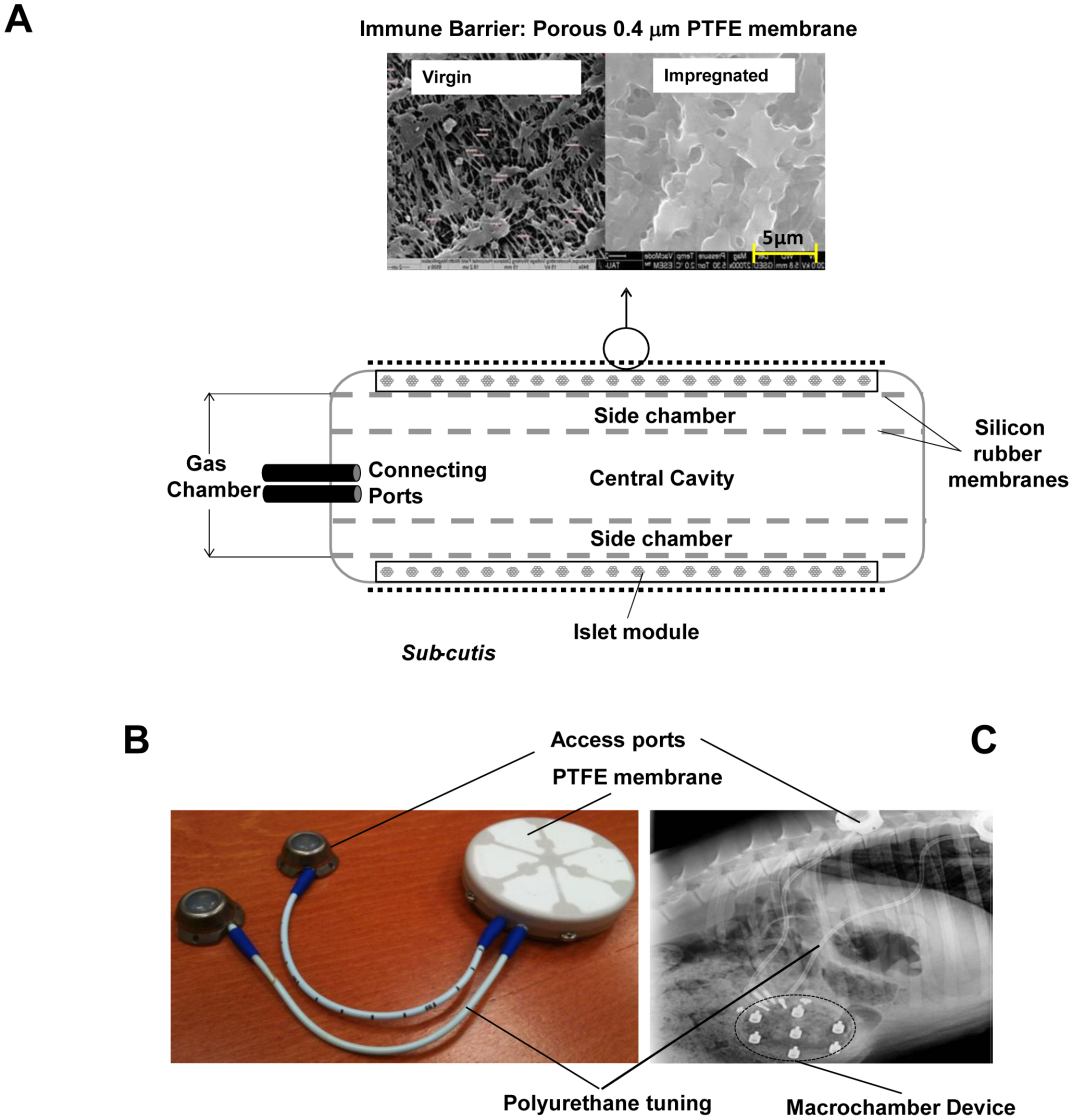
Chamber system for islet macroencapsulation. (**A**) Schematic view of the chamber composition. The core of the device is built as a gas module, connected to access ports for exogenous oxygen refueling. Active transport of solutes is achieved via a membrane impregnated with alginate (left, virgin; right, ready to use). Separated by gas permeable membranes, 2 compartments surround the central gas cavity that houses alginate-immobilized pancreatic islets. The plastic housing of the chamber has a latticelike design at both external surfaces and covered by hydrophylized PTFE porous membranes. (**B**) Photographic image of a completely assembled chamber with connected access ports. (**C**) X-ray image of an implanted recipient.

The subcutaneously implantable macrochamber is a disc-shaped device (diameter: 68 mm; thickness: 18 mm; **Fig. 1A**), composed of an islet module attached to a gas chamber. These modules are separated by 600 µm rubber silicon gas-permeable membranes (Nusil, Carpinteria, CA, USA) having surface area of 1,660 mm^2^ and porosity of 16%. The gas chamber is composed of 3 compartments. A 23 ml central cavity is separated from the 2-side chambers (4.4 ml each) by identical pair of silicone rubber membranes. It is connected by polyurethane tubes to 2 independent access ports (Smart Port; Angiodynamics, Latham, NY, USA) implanted subcutaneously and allowing for exogenous refueling of the gas chamber using a 27G transcutaneous needle. Gas mixture containing 1,011 mm Hg O_2_ (95% oxygen at 1.4 atm) and 53 mm Hg CO_2_ is daily refueled into the gas chamber. Oxygen is diffusing from the central cavity across the silicon membranes into the side gas chambers and towards the islet compartments where it dissolves in the aqueous environment of the alginate hydrogel, thus providing an adequate oxygen milieu.

The plastic housing of the chamber (polyether ether ketone [PEEK] Optima LT1R40; Invibio. Lancashire, UK) provides mechanical protection to the islet graft. The outside of the chamber consists of 2 connected layers of hydrophilized 0.4 µm porous PTFE membranes (Biopore, Millipore; Schwalbach, Germany) fixed onto the PEEK housing by medical epoxy adhesive (Billerica, MA, USA) to prevent immunologic communication between the recipient organism and the graft tissue (**Fig. 1B**). The double membrane was impregnated with alginate as follow: High mannuronic (HM) alginate (UP-HMW, Novamatrix; Sandvika, Norway) was dissolved in histidine-tryptophan-ketoglutarate (HTK) solution (Custodiol, Essential Pharmaceuticals Newtown; PA, USA) to a final concentration of 6% (w/v) and applied to the double Biopore membranes. The polymer was cross-linked with 30 mM BaCl_2_ in saline (Sigma; St. Louis; MO, USA). Excess barium was removed by 3 consecutive washing steps with 150 ml of 0.5 mM BaCl_2_ in saline-*N*-2-hydroxyethylpiperazine-*N*′-2-ethanesulfonic acid (HEPES) buffer (pH = 7.4). The impregnated membranes were lyophilized (Nextar; Ness Ziona, Israel) and sterilized by low-temperature ethylene oxide (ETO) cycle of 31°C for 18 h (Mediplast, Yavne, Israel) to minimize damage to the alginate. High guluronic acid (HG) alginate (UP-MVG, Novamatrix; Sandvika, Norway) used for islet immobilization was dissolved in HTK to a concentration of 0.5% and filter-sterilized through 0.45 µm membrane (Durapore, Millipore; Billerica, MA, USA). It was then lyophilized and sterilized by a certified service provider (Nextstar; Ness Ziona, Israel).

### Islet Isolation and Culture

Pancreata were obtained from 9–10 week old male Lewis rats weighing 260–280 g. Standard collagenase digestion was used with some modifications. Briefly, each pancreas was infused with collagenase (NB 8, Serva; Heidelberg, Germany) and bovine DNase (Sigma; St Louis, MO, USA) in Hank’s balanced salt solution (HBSS) for digestion. Islets were purified on a discontinuous Histopaque gradient (1.119/1.100/1.077/in RPMI) and washed and cultured for 5–7 days in complete CR medium (CMRL:RPMI medium [1∶1] supplemented with 10% fetal bovine serum [Biological Industries, Bet HaEmek, Israel]) before immobilization and integration into the chamber system. For quality control, oxygen consumption rates (OCR) were determined as previously described (by inserting a Clark-type electrode into an islets-containing chamber containing 8.3 mM glucose) [15].

### Islet Immobilization and Integration

Following islet counting and conversion to IEQ as previously described [15], samples of 60,000 to 70,000 IEQ were collected and gently mixed with 3.0% (w/v) sterile HG alginate dissolved in HTK and spread in the islet compartment of the chamber system. It was then cross-linked by applying flat Sintered glass (Pyrex; Staffordshire, UK) saturated with 70 mM strontium chloride plus 20 mM HEPES (pH = 7.4). The thickness of the alginate/islet slab was 600 µm and seeding density was 4,160±380 IEQ cm^2^.

### Islet Recipients

All minipig experiments were performed at the Institute for Animal Research (IAR; Kibbutz Lahav, Israel). Male, 5-month-old, Sinclair minipigs obtained from Harlan, Yoqneam, Israel (10–12 kg body weight [BW]) were used as recipient animals. The animals were fed with 2×250 g dry sow food (Ambar, Hadera Israel) daily (at 9 am and 3 pm). For diabetes induction, a catheter was implanted into the external jugular vein. STZ (Sigma-Aldrich; St Louis, MO, USA) was diluted in cold citrate buffer (pH = 4.5) immediately before use and injected via the jugular vein for 3 consecutive days at doses of 120, 100 and 100 mg/kg BW, respectively. Diabetes was determined by fasting blood glucose >300 mg/dl in 3 consecutive days or more. Animal weight was recorded twice weekly throughout the experiment.

### Implantation Procedure

Animals were fasted overnight with free access to water before transplantation. Azaperon (15 mg/kg intravenously [i.v.]; Cilag-Janssen; Neuss, Germany), atropin (0.2 mg/kg i.v.; Braun; Melsungen, Germany), and ketanest (3 mg/kg i.v.; Ratiopharm; Ulm, Germany) were administered for premedication. Anesthesia was maintained with isoflurane (Abbott; Wiesbaden, Germany) and the ventilation was performed via a customized mask. A 5–8 cm incision was made in the lateral abdomen and a subcutaneous pocket was bluntly dissected. Two additional small incisions were made on the back of the animals for implantation of the oxygen ports. The chamber and the port system were implanted and connected via subcutaneous tunneling of polyurethane tubes. The skin was sutured intracutaneously.

### Animal Follow Up

After graft implantation, the first group of animals were followed for 30 days (n = 5) before retrieval of the device and were monitored for additional 3 days post explantation by clinical assessment and daily blood glucose measurements using a commercial glucometer (AccuChek Aviva, Roche Diagnostics; Hod HaSharon, Israel). One of the animals carried a continuous glucometer (iPRO, Medtronic Minneapolis USA) for a week. The second group of animals (n = 3) were transplanted with equal islet mass and followed up until hyperglycemia resumed. For determination of glucose-stimulated insulin release, an i.v. glucose tolerance test (ivGTT) was performed in diabetic animals before transplantation, 2 weeks after transplantation, and 5 days post explantation of the graft-containing chamber system. Glucose was infused at a concentration of 500 mg/kg BW and blood glucose was measured at time 0, 10, 30, 60 and 120 min following glucose infusion. Glucose was measured by commercial glucometer. Rat C-peptide was assayed in pig serum samples, after overnight fasting, in 5-day intervals throughout the observation period using enzyme-linked immunosorbent assay (ELISA) method according to manufacturer’s instructions (ALPCO diagnostics; Salem, NH, USA).

### Oxygen Gas Measurements

Measurement of oxygen concentration in the gas compartments was performed daily, just before the routine refueling. Animals were sedated with isofluran, a 27G syringe needle was inserted into one of the implanted ports, and a 250 µl sample was taken from the gas chamber. Oxygen concentration was then determined using a Clark-type electrode following pre-calibration at 0 and 570 mm Hg.

### Immunohistochemistry

Immunohistochemistry experiments were performed in the lab of P. de Vos. For immunohistochemical assessment, explanted islet slabs were dehydrated, processed in paraffin, and sectioned at 4 µm. Mouse anti-rat IgG1 insulin (Sigma; St Louis, MO, USA) was used as the primary antibody and anti-mouse IgG1 horseradish peroxidase (HRP; Southern Biotechnology Associates Inc; Birmingham, AL, USA) was used as the secondary antibody. The signal was visualized using avidin-biotin labeling and 3,3′-diaminobenzidine (Invitrogen; Camarillo, CA, USA). All slides were counterstained with Mayer’s hematoxylin. Isotype control antibody was used to confirm staining specificity.

### Detection of Porcine DNA Penetration

The experiments were performed in the lab of E.C. Lewis. Samples were taken by trans-membrane puncturing. DNA was extracted using standard methods and polymerase chain reaction (PCR) was carried out using XP cycler technique (BIOER; Hangzhou, China) with the following genome-targeted primers: porcine-actin forward 5′-TGTTCGAGACCTTCAACACG-3′ reverse 5′-CAGCTTCTCCTTGATGTCCC-3′. Reaction mixture included PCR ReadyMix™ (Sigma-Aldrich; St Louis, MO, USA) and Taq polymerase (Red Load Taq master, Larova; Teltow, Germany). For positive controls, we used extracts of porcine muscle, liver, and spleen containing 10 ng of DNA and analyzed these samples in parallel to the intrachamber samples. The sensitivity of the test, as reported by the manufacturer, is 10 pg/ml.

### Detection of Anti-rat Immunoglobulin

The experiments were performed in the lab of E.C. Lewis. Rat pancreas was lysed in radio immunoprecipitation assay (RIPA) lysis buffer and 96-well flat bottom ELISA plates were coated with 10 ng/ml of rat pancreatic protein in phosphate-buffered saline (PBS) at a total volume of 100 µl for 2 h at 25°C. Wells were then blocked with 1% horse serum (VECTOR laboratories; Burlingame CA, USA) in a total volume of 300 µl PBS containing 0.05% Tween 20 (TPBS), washed with TPBS and incubated with pig serum containing 1% normal horse serum for 2 h. Following washing steps, biotin-conjugated goat anti-swine IgG antibody (Jackson immunoresearch, West Grove; PA, USA) was added at a concentration of 200 ng/ml for 2 h. After repeated washing, HRP (1∶1,000 dilution; R&D Systems; Abingdon, UK) was added for 20 min. For detection, 3,3′,5,5′- tetramethylbenzidine (TMB) substrate (Southern Biotech; Birmingham, AL, USA) was added for approximately 10 min after which the reaction was stopped using 100 µl of HCl 0.5 M. Light absorbance was determined at 450 nm using an ELISA plate reader (BioRad; Hercules CA, USA). Positive control serum was obtained from pigs that were sensitized with rat protein by intramuscular (i.m.) injection of rat islets lysate (4,000 IEQ each at 2-week intervals) and complete Freund’s adjuvant (CFA) (Sigma; St Louis, MO, USA).

### In-vitro Determination of the Diffusion and Permeability Capacities of the Membrane System

Impregnated PTFE membranes were installed between 2 diffusion cells (Permegear Inc; Hellertown, PA, USA). Index-molecules were added on one side and samples were taken on the opposite side for up to 48 h. Naïve, non-impregnated, membranes were tested in parallel. To determine the permeability of the membrane for components of the immune system, mouse IgG (Jackson; Newmarket, UK) and human complement C1q (AbD Serotec; Dusseldorf, Germany) were diluted in 0.5 mM BaCl_2_ in saline and 13 mM HEPES (pH = 7.4) to final concentrations of 135 and 160 µg/ml, respectively, and loaded into the source side of diffusion chamber. Immediately and every 30 min thereafter, samples were taken from the sink side across the membrane and tested for the solutes concentration. Briefly, 96-well plates (Corning; Tewksbury, MA, USA) were coated with goat anti-mouse IgG or with goat anti-human C1q (Jackson; Newmarket, UK) in PBS. Blocking solution was 1% bovine serum albumin (BSA). Peroxidase mouse anti-goat IgG (Jackson; Newmarket, UK) was used as conjugate and TMB (Cayman Chemicals; Ann Arbor, MI, USA) as substrate. Light absorbance was determined at 450 nm using an ELISA plate reader (BioTek instruments; Winooski, VT, USA). To determine the diffusion of insulin through the membrane, human insulin (0.1 mg/ml, Sigma; St Louis, MO, USA) was dissolved in 4% BSA and introduced to the source side of the diffusion chamber. Insulin concentrations across the membrane were determined using insulin ELISA (Mercodia; Uppsala, Sweden) according to manufacturer’s instructions. Diffusion of glucose was determined by adding glucose at a concentration of 5.5 mM dissolved in 4% BSA. Samples from the opposite side of the diffusion chamber were analyzed using a commercial glucometer (Accu-Check, Roche Diagnostics; Hod HaSharon, Israel). The ability of live viral particles to cross the membrane was tested using pseudo-typed lentiviral particles expressing a reporter ZGreen gene product. These experiments were performed in the lab of R. Taube. Viral production and infection protocols were performed according to Kuzmina et al [16]. Briefly, Vesicular Stomatitis Virus glycoprotein (VSV-G) lentiviral particles expressing a reporter green fluorescent protein (ZsGreen) gene were generated by co-transfection of HEK293T cells with human immunodeficiency virus (HIV) packaging plasmids (Tat;Rev;Gag Pol) and the cytomegalovirus (CMV)-ZsGreen expression lentiviral vector. At 48 h post transfection, supernatant containing viral particles was collected, filtered, and concentrated by ultracentrifugation. MRC-5 primary human fibroblasts were seeded on 24-well plates (Transwell, Corning; Tewksbury, MA, USA) at a density of 60,000 cells/well. Each well included a transwell, which was separated from the well by PTFE double membrane. Transwells were inoculated with high titer HIV-CMV-Zs-Green at titer of 6.0×10^4^ to 9.6×10^5^ (1–16 multiplicity of infection [MOI]). To quantify infection rate, ZsGreen expression was analyzed by 72 h post infection by immunofluorescence and fluorescence-activated cell sorting (FACS) analyses. As a control, the culture experiment was conducted without the membrane system separating the cells from the virus.

### Data Analysis and Statistics

All results are presented as mean ± standard deviation (SD). T-test was used to evaluate the difference in blood glucose levels in the pre-implantation period, implantation period, and post-implantation period. Results were considered statistically significant at *P*<0.025. Diffusion rates of glucose and insulin were calculated according to Fick’s law.

## Results

### Assessment of Macroencapsulated Islet Graft Function

The potency of macroencapsulated islet grafts to reverse diabetes was determined in a large xenogeneic animal model. STZ-induced diabetic minipigs were transplanted with rat islet grafts contained in the macrochamber system and implanted under the skin (**Fig. 1**). To ensure comparable islet quality, OCR was determined for all islet preparations before integration into the device. Animal and graft characteristics are listed in Table 1.

**Table 1:** Characteristic of transplant recipients and islet grafts.

Animal-ID	IEQ	BW (kg)	IEQ/kg	OCR (pmoles/min/IEQ)	Total OCR(pmoles/min)	Follow-up (days)
#1548	67,600	10.85	6,230	3.65	246,740	30
# 1549	70,400	11.15	6,314	4.25	299,200	30
# 1593	58,000	7.90	7,342	4.13	239,540	30
#1594	69,100	10.3	6,708	3.90	269,440	30
#1674	56,800	8.05	7,056	3.34	189,710	30
#1709	57,600	8.9	6,472	3.25	187,200	85
#1736	66,000	9.65	6,839	2.88	190,080	85
#1756	57,600	10.5	5,486	3.06	176,260	85
**Average**	**62,890**	**9.7**	**6,556**	**3.56**	224,770	
**SD**	**5,900**	**1.3**	**572**	**0.50**	45,400	

BW, body weight; IEQ, islet equivalents; OCR, oxygen consumption rate; SD, standard deviation.

Upon diabetes induction, animals showed a rapid weight loss (25±10% loss by 14 days after the last STZ injection). At time of implantation, the average weight of the recipients was stabilized at 9.5±1.25 kg (**Table 1**), about 3% above the minimal weight level. Following transplantation and corresponding to blood glucose normalization, all animals showed a steady increase in body mass.

**Figure 2:**
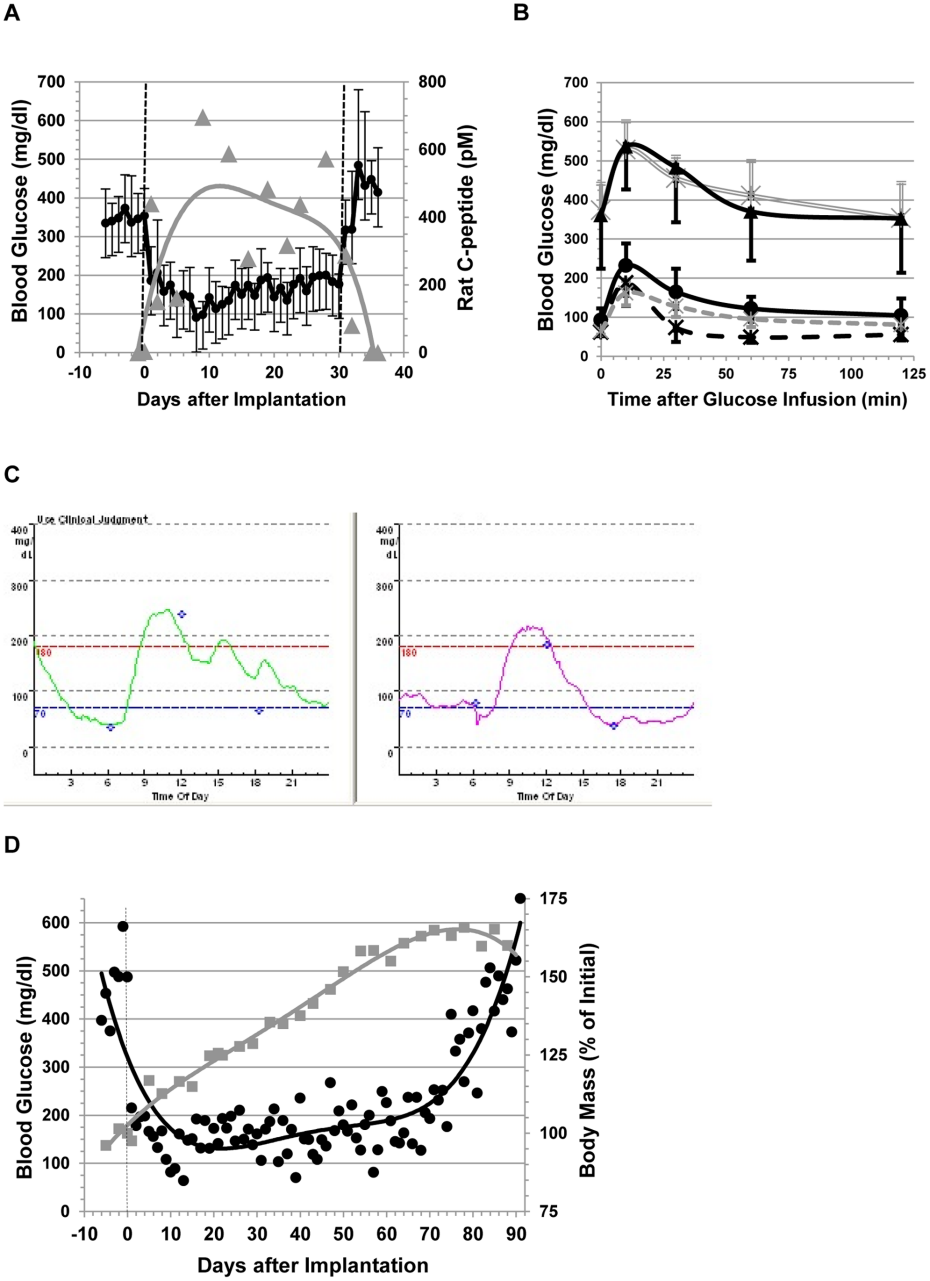
Function of macroencapsulated rat islet grafts. STZ-induced diabetic minipigs were transplanted with 6,730±475 IEQ/kg BW of rat islets immobilized and integrated into the macrochamber system. ivGTT was performed prior to graft implantation, 2 weeks after implantation, and after retrieval of the graft. BW was recorded daily throughout the observation period. (**A**) Fasting blood glucose levels (black) of group one transplanted minipigs (n = 5). The graft was removed on day 30 and hyperglycemia recurred demonstrating that graft function was responsible for normoglycemia during the implantation period. Rat C-peptide levels (grey) are presented as 4^th^ grade polynomial curves. Error bars represent SD. *P*<0.001 for comparing glucose levels during the implantation period vs the pre- and post-implantation periods (t-test). (**B**) Blood glucose levels during ivGTT of transplanted animals at two weeks (black circles) and after retrieval of the graft-containing device (black triangles). Diabetic minipigs (n = 24; double grey), naïve healthy mini-pigs (n = 11; black dashed) and naïve healthy rats (n = 36; grey dashed) served as controls. Error bars represent SD. *P*<0.001 for comparing AUCs (diabetic minipigs) in the implantation period vs the pre- and post-implantation periods (t-test). (**C**) Two-day continuous glucose monitoring records of an implanted animal during week 2. (**D**) Fasting blood glucose levels (black) of group 2 transplanted minipigs (n = 3) and corresponding BW (grey). Normoglycemia was achieved rapidly after transplantation and was retained until the BW increased to a critical level of >160% of the initial BW. Data are presented as 5^th^ grade polynomial curves. *P*<0.001 for comparing glucose levels during the first 75 days of the implantation period vs the pre-implantation period (t-test).

For the first group of animals that was followed for 30 days, the chamber implantation resulted in a stable reversal of diabetes throughout the observation time (**Fig. 2A, B, C**). After removing the islet-graft-containing chamber on day 30, all animals showed an immediate recurrence of hyperglycemia (**Fig. 2A, B**). The average fasting blood glucose levels during the implantation period were significantly different than those in the pre- and post-implantation periods (**Fig. 2A**; *P*<0.001; t-test). Implanted animals demonstrated ivGTT profile, which was similar to that of healthy rat donor animals and slightly delayed compared to the minipig profile. Area under the curve (AUC) calculated for healthy rats and for pigs implanted with rat islets were different but close (12,600±1,950 and 16,700±4260, respectively) and very different from AUC calculated for hyperglycemic animals (51,100±7380 and 56,450±14,360 for diabetic animals before implantation and following explantation of the macrochambers, respectively) (**Fig. 2B**; *P*<0.001 for comparing AUCs for the implantation period vs the pre- and post-implantation periods in diabetic minipigs; t-test). In the second group of animals with prolonged follow up (**Fig. 2D**), the graft was explanted only after the animals resumed a diabetic state. Fasting blood glucose levels in the first 75 days of the implantation period differed significantly from those in the pre-implantation period (**Fig. 2D**; *P*<0.001; t-test). The animals in the second group presented a massive BW gain. By 2 months after implantation, at the time of diabetic recurrence, their weight was >160% that of the original weight, and therefore the dose of the contained islets, calculated based on actual implanted grafts with assumption of 100% viability, decreased to <4,000 IEQ/kg, suggesting that at such a dose, the device could no longer maintain normal blood glucose levels (**Fig. 2D**).

Serum levels of C-reactive protein (CRP) in implanted animals were within normal range before implantation (2.2±0.86 mg/dl), peaked to 12.0±6.13 mg/dl within 2–5 days after implantation and declined to 3.6±1.25 mg/dl within 10 to 15 days, suggesting that no prolonged systemic inflammation was associated with the macrochamber implantation. Blood markers that were followed also responded to induction of diabetes and to the device. Table 2 demonstrates that several blood markers were adversely affected by the diabetic state and returned to near normal levels within 2 weeks of implantation.

**Table 2:** Blood markers in healthy, diabetic and device carrying mini-pigs.

Marker	Before	Diabetic	Implanted
**SGOT (U/l)**	36±12	168±163	50±19
**Total-bilirubin (mg/dl)**	0.07±0.04	0.99±0.96	0.21±0.16
**Cholesterol (mg/dl)**	70±17	164±173	78±21
**Triglycerides (mg/dl)**	31±18	387±455	34±21
**Urea (mg/dl)**	22±6	42±8	28±5

Data presented are mean ± standard deviation.

SGOT, serum glutamic oxaloacetic transaminase.

### Oxygen Concentration in the Gas Chamber

**Figure 3:**
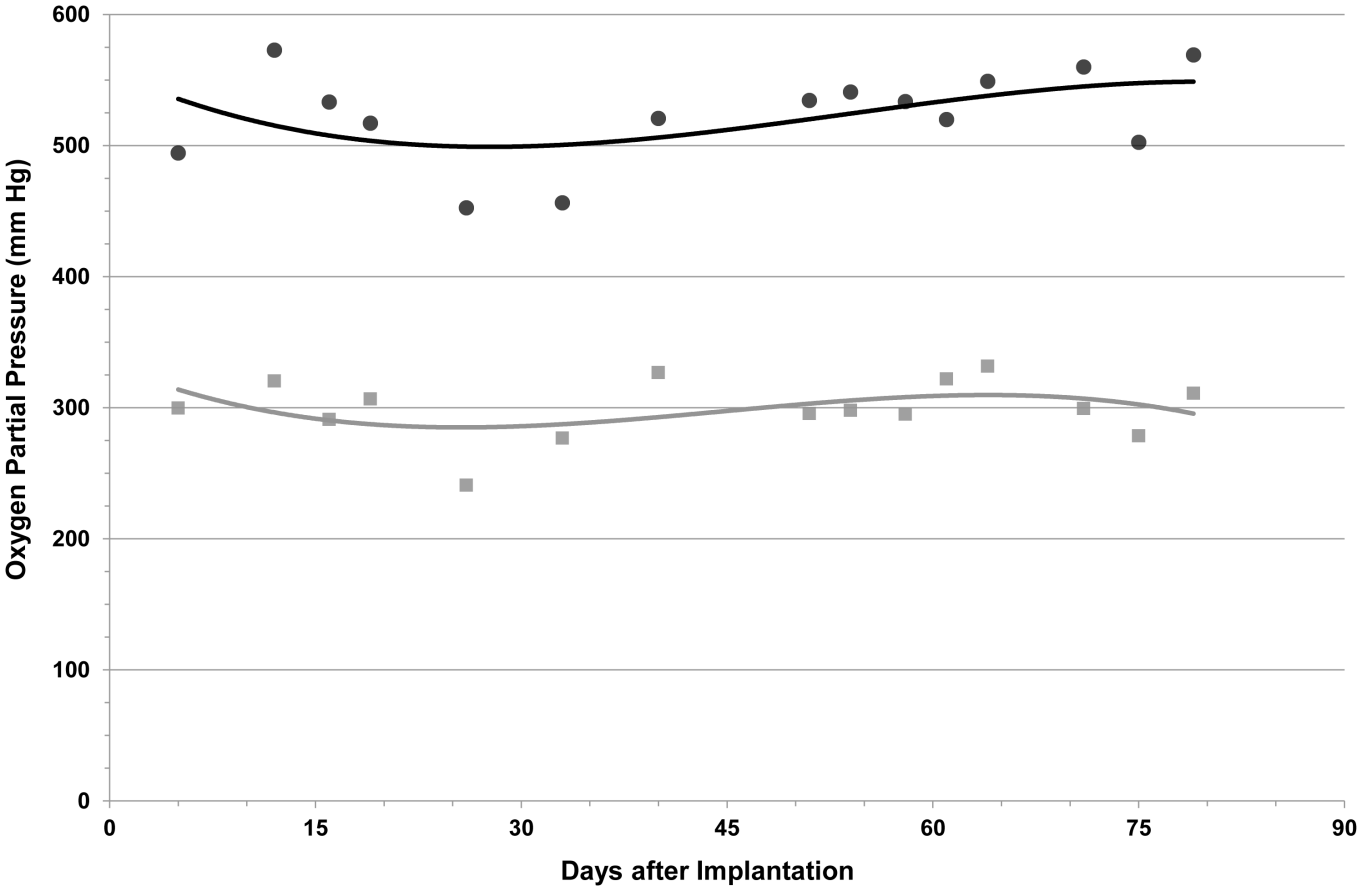
Oxygen partial pressure in the gas chamber. Levels of oxygen at the end of successive 24 h cycles were monitored in the central cavity and in the 2 side chambers. Solid black line, central cavity; solid grey line, side chambers. Data are presented as the 3^rd^ grade polynomial curves.

The gas blend injected into the central cavity raised oxygen partial pressure to a zenith of 1,011 mm Hg. During the subsequent 24 h it was steadily consumed by the islet graft and lost by diffusion into the neighboring host tissue. Oxygen concentrations in the central cavity and in the 2-side chambers decreased to nadirs of 524±36 mm Hg and 343±37 mm Hg, respectively (**Fig. 3**). In other words, oxygen concentration at the gas chamber-islet graft boundary was constantly kept at >300 mm Hg. Inside the islet graft module, another oxygen concentration gradient was being formed as the islet graft–tissue boundary was characterized by venous-type oxygen concentration of only approximately 38 mm Hg. This gradient drove oxygen across the hydrogel slab and ensured adequate levels of oxygen to every contained islet.

### Evaluation of Islet Grafts after Explantation

**Figure 4:**
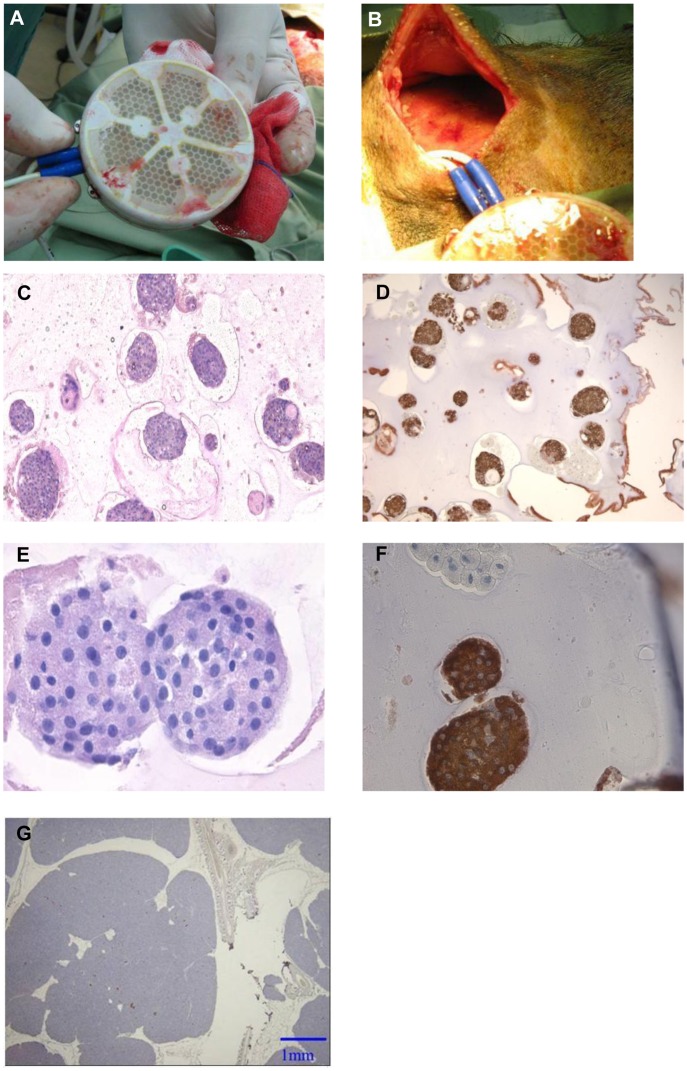
Morphologic assessment of recipient pancreas and pancreatic islets following 90 days of implantation period. (**A**) A device after explanation. (**B**) The tissue surrounding the device at explantation. (**C–F**) Representative images of alginate/islet slabs (C,D - 10×; E, F - 40×). Left: HE; islets displayed an intact structure and no signs of disintegration within the alginate. Right: Immunohistochemistry for insulin showed intense cytoplasmic staining as typically seen in intact rat islets. (**G**) Representative image (2×) of a pancreas of a recipient animal at autopsy.

At explantation, the device looked intact and no inflammation was detected at the implantation site; the device was surrounded by a thin (1-mm thick) well-vascularized fibrotic tissue (**Fig. 4A, 4B**). Morphologic evaluation of islet grafts was performed on alginate/islet slabs that were explanted after 90 days (n = 3). Hematoxylin and eosin (HE) staining showed intact islet structures, which were not different from those in freshly isolated rat islets. The islet graft exhibited intense insulin staining (**Fig. 4C–4F**), indicating continuing survival and function of the islets. This functionality together with the observed diabetic state of the animals suggests, as mentioned above, that the recurrence of the diabetic state was due to a decreased dose of IEQ/kg resulting from an increased BW and not because of a deterioration of the islet graft. In parallel, only a few insulin positively stained scattered cells were observed in pancreata of explanted recipients.

### Evaluation of Cellular/immune Barrier Function of the Chamber System

**Figure 5:**
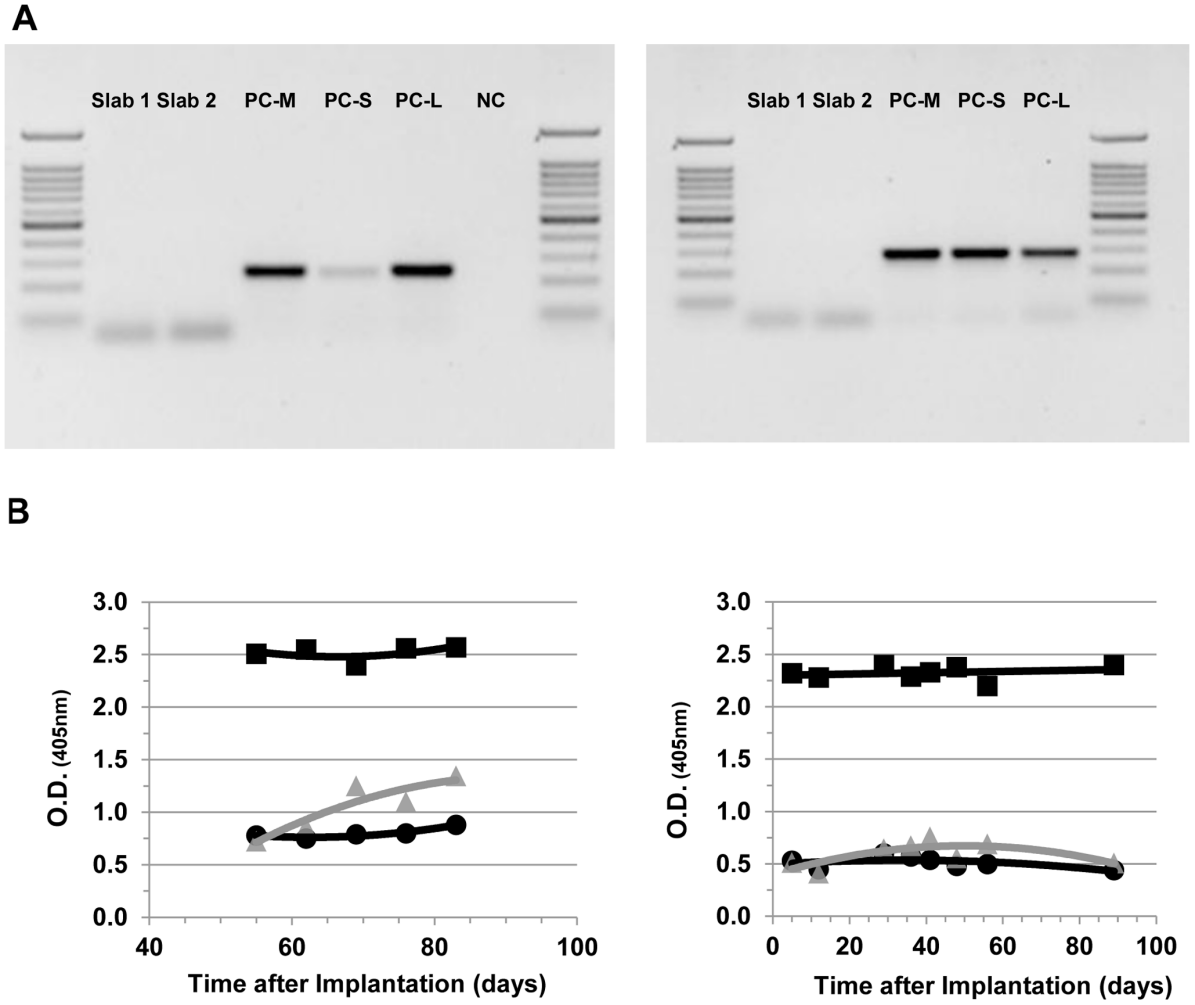
Determination of the barrier function of the chamber system. (**A**) Biopsies from alginate/islet slabs were taken at 30 days (left panel, slabs 1 and 2) and at 90 days (right panel, slab 3) to test for porcine DNA contamination inside the chamber. At both time points, no porcine DNA was detectable in any of the samples. Non-tissue samples were used as negative controls (NC). Tissue extracts from porcine muscle (M), spleen (S), and liver (L) served as positive controls (PC). (**B**) Serum samples of rat islet graft recipients were taken at various time points throughout the observation period of up to 90 days to test for development of anti-rat immunoglobulin. The 2 graphs show data of 2 individual recipients (left, 1709; right, 1736). Squares: positive control (sensitized animal); circles: negative control (healthy animal); triangles: diabetic minipig transplanted with encapsulated rat islets.

In order to test whether the various membrane layers constitute an adequate cellular barrier, alginate/islet samples were assessed for porcine DNA at 30 and 90 days post implantation. As shown in Fig. 5A, no porcine DNA contamination was detected at both time points. In parallel, no CD3-positive staining was detected (data not shown).

The elution of rat proteins from inside the chamber to the recipient was tested by evaluating anti-pancreatic rat antibodies in minipig serum. Whereas animals sensitized for rat protein (positive controls) exhibited persistent circulating anti-rat IgG, the recipients of macroencapsulated rat islet grafts were not sensitized even after a prolonged exposure of up to 90 days (**Fig. 5B**).

### Assessment of Diffusion Capacity of the Membrane System

As indicated by calculation of the diffusion coefficients, the permeability of the reconstituted membrane to glucose was very similar to that of a plain membrane (diffusion coefficients: 1.69±0.01 and 1.37±0.31 [cm^2^/sec]×10^−6^, respectively). The permeability of the reconstituted membrane to insulin was slightly impaired compared to that of a plain membrane barrier (diffusion coefficient: 1.11±0.42 vs 2.38±0.37 [cm^2^/sec] ×10^−7^).

**Figure 6:**
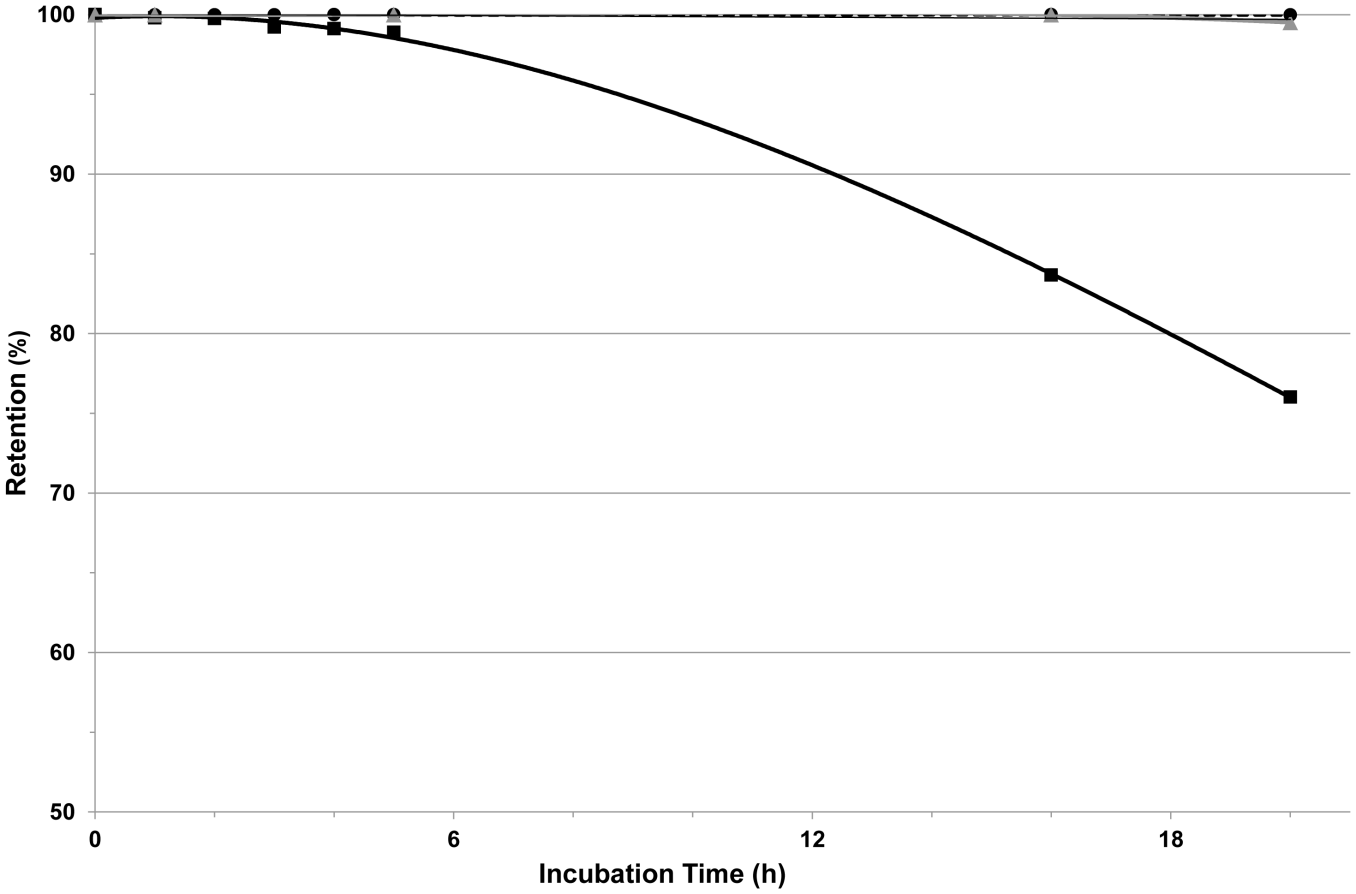
Diffusion of large molecules across the membrane system is impeded. Diffusion of mouse IgG across naïve membrane (squares) or diffusions of IgG (circles) or C1q (triangles) across reconstituted membranes. Values represent the relative amount of solute retained in the loaded chamber as a function of time.

Permeability of large index molecules (mouse IgG and human complement C1q) across the membrane system was measured as an indicator of the capacity of the barrier to protect an islet graft against the host humoral arm of immune rejection. For both IgG and C1q, diffusion across the membrane system was markedly impeded; more than 99.5% of the original loads were retained behind the barrier after 20 h (**Fig. 6**).

**Figure 7:**
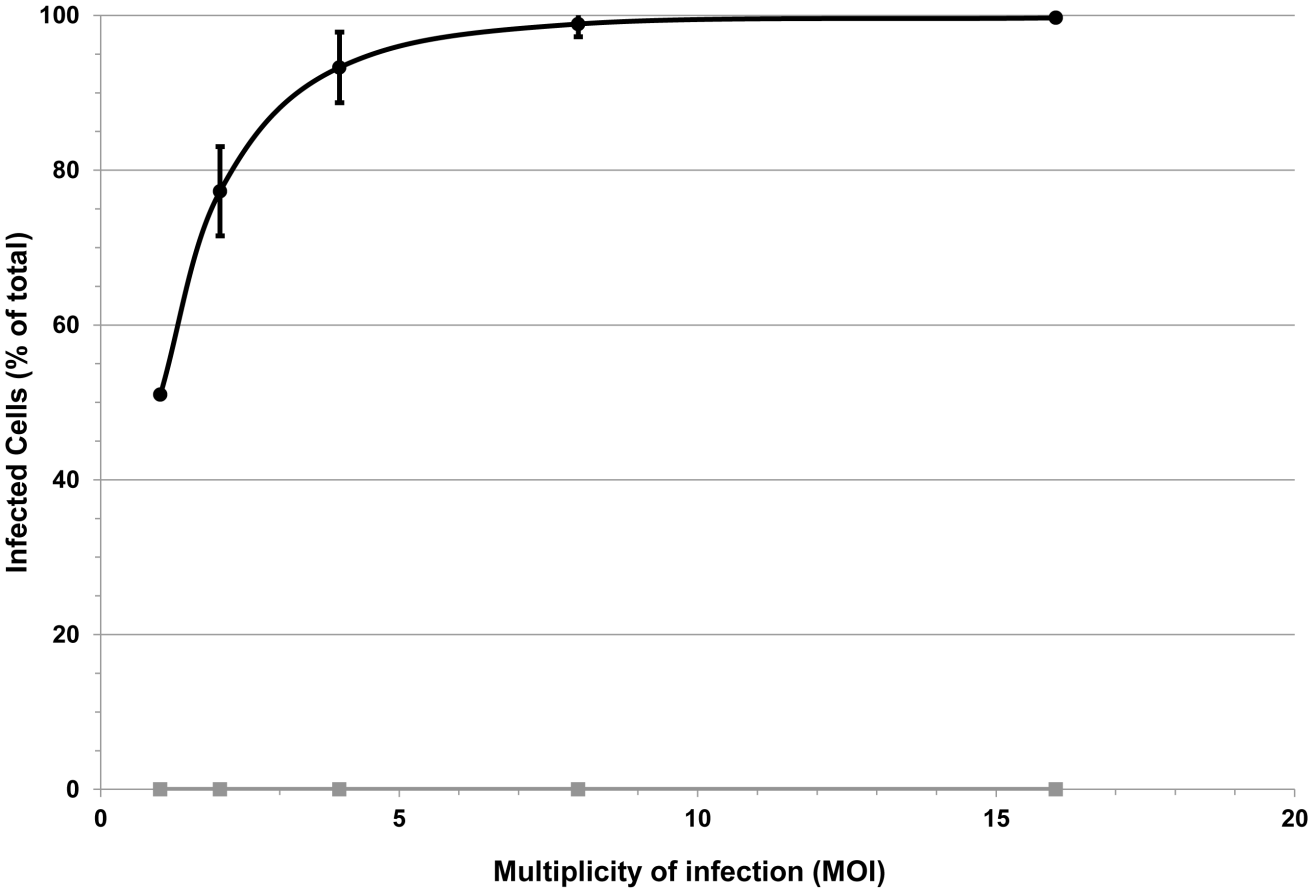
Barrier function of the membrane system for viral transmission. MRC-5 primary fibroblasts cultured in 24-transwell plates were separated from suspension containing reporter Lentiviruses by the membrane system for a period of 72 h. Infection rates were quantified by FACS analyses. Regardless of the MOI level, no relevant infection was detected in the membrane group (squares). In the control group (naïve membrane; circles), infection levels corresponded to increasing MOI levels from 40% up to 99.5%.

Finally, we evaluated the ability of the barrier function of the chamber system to prevent viral transmission, by using the membrane system to separate cultured primary human fibroblasts and ZsGreen reporter pseudotyped lentivirus. As shown in Fig. 7, regardless of the MOI levels, the infection rates in the reconstituted membrane group was below the detection level, suggesting sufficient barrier function. In contrast, cells separated by plain membranes showed MOI-dependent infection rate achieving almost 100% at MOI of 16.

## Discussion

The objective of this study was to evaluate, in a clinically relevant diabetes model, a system of islet macroencapsulation that addresses major obstacles in current islet transplantation and opens up a strategy for safe clinical application of allo- and potentially xeno-islet transplantation.

The major impediments to a successful and more widespread application of islet transplantation are the persistent shortage of donor organs, and the chronic need for immunsuppressive therapy to control allo- and autoimmunity [17], [18]. The standard procedure of islet transplantation usually requires intraportal infusion of isolated islets from more than one donor organ to achieve insulin-independence, due to massive loss of infused islets [19], [20]. Various stress factors are responsible for this loss, including inflammatory reactions at the transplant site, exposure to oxidative stress, and chronic hypoxia of the islet graft due to the initial severing from the original vascular bed and insufficient revascularization [21]–[24]. To minimize immunologic reactions against the allo-islet graft, a potent induction therapy and immunosuppressive maintenance regimen is required. The associated side effects and long-term risks are highly relevant and necessitate careful patient selection [25]. Encapsulation of islets in a semi-permeable membrane can provide an alternative approach. In particular, microencapsulation has been shown to be feasible in various experimental systems [23]–[25] and in pilot clinical trials [26]. However, the limited lifetime of microencapsulated islets and the large islet tissue volume required are obstacles that must be overcome to make this approach clinically viable. Analyzing oxygen supply as a limiting factor, Dulong and Legallaise developed a mathematical model that clearly demonstrates the physiological limitations of microencapsulated islets when transplanted into a site with venous-type oxygen tension. The model also predicts that encapsulation of islets in flat sheet geometry provides a better environment for survival and clinical functioning [26]. A pivotal experiment demonstrated the feasibility of the flat sheet encapsulation procedure to cure diabetes in dogs. However, surface density of the encapsulated islets was low [27].

As previously described [14], [15], [28], we have developed a planar chamber system for safe islet implantation. It consists of an oxygen supply module and 3-layer immune barrier. The gas module provides adequate amounts of oxygen to the graft in a poorly oxygenated environment. The macrochamber device–shown to protect a xenogeneic islets graft from rejection for a minimum of 90 days–is constructed as a rigid plastic housing and contains hydrophilized PTFE membranes and 2 types of alginate hydrogels. The central cavity of our device is refueled with oxygen at partial pressure of 1,011 mm Hg (>6 times its atmospheric pressure). At no time during the 24 h interval between successive oxygen refueling, was this pressure reduced <500 mm Hg at the central cavity and <300 mm Hg at the gas chamber-islet module interface. The steep gradient ensures steady delivery of oxygen to the encapsulated islets, which always enjoy permissive oxygen tension requested for maximal insulin secretion. This environment probably also improves islet viability and functionality. Histological examinations of explanted grafts suggested that long-term exposure of islets to supraphysiological level of oxygen did not have a cytotoxic effect, a finding which is consistent with prior studies [15]. Unpublished data from our lab demonstrated that only lengthy exposure (>week) to oxygen levels ≥75% led to graft malfunctioning, but with the current 3-compartment gas-chamber design (central cavity and 2 side chambers), maximal levels of oxygen in the islet module-side chamber boundary were always kept at <70% and even this, for only a short period of time (<2 h/day) (unpublished data).

The device evaluated in the current study is an improved version of the previously described device that was assessed in a rat model of diabetes [15], as follows: i) increased iselt biomass as the diameter of the current device is larger (68 mm vs 31 mm in the previous prototype), surface density of the islets doubled, and it has 2 islet modules (one at each surface), allowing it to carry up to 150,000 IEQ (vs <3,000 in the previous prototype); ii) better gas ventilation with a 3-compartment design (a central cavity and 2 side chambers) that allows refueling with highly concentrated oxygen (95%) at moderate pressure (up to 1.4 atm) into the central cavity (vs 1-compartment design and refueling with a mixture of 40% oxygen and 5% CO_2_ at atmospheric pressure in the previous prototype); and iii) an improved immune barrier with an alginate-impregnated double membrane that is impermeable to IgG.

In immunoisolation, the xenogeneic setting is considered to be more challenging than the allogeneic setting as the former is characterized by the occurrence of both direct and indirect cellular immune responses whereas in the latter, predominantly direct immune reactions (humoral immune response) can also be activated. The direct response is dependent on the presentation of foreign antigens to the host immune system and is therefore dependent upon cell-to-cell contacts, which can be prevented by a plain membrane barrier [9]. The indirect response, which is activated by soluble donor antigens that activate the humeral immune system or are being picked up by migrating antigen presenting cells (APCs), occurs predominantly in xenotransplantation. This response is referred to as a delayed-type hypersensitivity response and has been considered as the Achilles heel of immunoisolated xenotransplantation for years [10]. Current membranes cannot prevent this response as the immune barrier must meet more stringent requirements. Our membranes were not designed to prevent the response but rather to protect against it. Therefore, we designed a multilayer membrane that proved to be impermeable for IgG with a molecular weight [MW] of 160 kDa. As xenoreactive antibodies are not crucial for islets xenograft rejection [29], complement activation may take the lead. However, the pivotal complement factor C1q (MW∼410 kDa) is also retained by the membrane and the molecular mass of IgM (MW∼950 kDa), which may also drive rejection is even larger.

For creating an immune barrier, islets are first immobilized in a flat sheet of HG alginate. The second barrier is based upon hydrophylized PTFE membrane, which prevents cell-to-cell contact. The pores of this membrane are then sealed following impregnation with HM acid alginate, which represents the third layer. Our findings demonstrated that this combined barrier structure allowed for free diffusion of glucose and only marginally attenuated insulin diffusion. In contrast, permeability of both IgG and C1q was almost totally prevented. Therefore, we hypothesize that immune reactions against the graft are sufficiently prohibited. The ability of the system presented herein to restore and maintain normoglycemia in diabetic minipigs, the absence of contaminating pig DNA inside the chamber system, and the absence of anti-rat antibodies in the recipient provide further evidence that the macrochamber has the potential to evolve into a successful and safe clinical application in a xenotransplant setting.

Five animals were transplanted with islets contained in a macrochamber. Judged by systemic CRP, no inflammation accompanied the surgery. Animals showed a rapid and persistent normalization of glycemic control and levels of several blood markers were also normalized. The restoration and maintenance of normoglycemia due to islet graft function was demonstrated by islet-deficient pancreata of recipients and recurrence of the diabetic state upon removal of the graft-bearing chamber. In addition, rat C-peptide was detectable during the implantation period and disappeared from minipig serum immediately after explantation. A second group of animals were followed for a prolonged time period. In these animals, the BW increased by 65% over 70 days period following transplantation. Although the initially transplanted islet mass of 6,270±700 IEQ/kg BW is approximately 20% of islet mass reported in other xenotransplant models [6], it was effective in restoring glycemic control in the recipient animals. However, as BW steadily increased, the nominal dose decreased and eventually impacted the blood glucose levels. Histological and immunohistochemical analyses showed that the recipient’s pancreas was almost depleted of beta-cells and that islet graft was morphologically intact with intense insulin staining. Therefore, graft failure was not likely a consequence of islet regeneration, immune rejection, or insufficient nutrient/oxygen supply.

Xenotransplantation using pig islets is considered to be a potential solution for the shortage of human organs and for the treatment of diabetes [30]. The designated pathogen free (DPF) status of donor pigs, however, cannot be attained for endogenous viruses, such as porcine endogenous retrovirus (PERV) as they are integrated into the genome. Despite intensive research, preclinical studies were unable to demonstrate PERV or PERV/C transmission to human cells [31]. Nevertheless, the immune barrier described herein adds another layer of confidence to the safety in xenotransplantation of islets.

For humans with type 1 diabetes (in whom a total of 300,000–500,000 IEQ is required for glycemic control), implantation of 2 devices with the dimensions of the devices described herein (albeit, with higher surface density) may be required. Another potential approach includes the implantation of an elliptically-designed device (18×110×70 mm in size) containing 500,000 IEQ.

In summary, we have demonstrated persistent restoration of normoglycemia in diabetic minipigs upon implantation of macroencapsulated xenogeneic islets without immunosuppressive therapy. The macrochamber, which provides substantial immune barrier and sufficient oxygenation, opens up a novel opportunity for clinical porcine islet xenotransplantation by alleviating the need for immunosuppression, and could lead to a substantial progress in the field of islet transplantation, and in particular, xenotransplantation.

## References

[1] Barton FB, Rickels MR, Alejandro R, Hering BJ, Wease S, et al (2012) Improvement in outcomes of clinical islet transplantation: 1999–2010. Diabetes Care 35: 1436–1445. 22723582 10.2337/dc12-0063 PMC3379615

[2] Thompson DM, Meloche M, Ao Z, Paty B, Keown P, et al (2011) Reduced progression of diabetic microvascular complications with islet cell transplantation compared with intensive medical therapy. Transplantation 91: 373–378. 21258272 10.1097/TP.0b013e31820437f3

[3] Bellin MD, Barton FB, Heitman A, Harmon JV, Kandaswamy R, et al (2012) Potent induction immunotherapy promotes long-term insulin independence after islet transplantation in type 1 diabetes. Am J Transplant 12: 1576–1583. 22494609 10.1111/j.1600-6143.2011.03977.x PMC3390261

[4] CITR Research Group (2009) 2007 update on allogeneic islet transplantation from the Collaborative Islet Transplant Registry (CITR). Cell Transplant 18: 753–767. 19796497 10.3727/096368909X470874

[5] Ludwig B, Ludwig S, Steffen A, Saeger HD, Bornstein SR (2010) Islet versus pancreas transplantation in type 1 diabetes: competitive or complementary? Curr Diab Rep 10: 506–511. 20830612 10.1007/s11892-010-0146-y

[6] Dufrane D, Goebbels RM, Saliez A, Guiot Y, Gianello P (2006) Six-month survival of microencapsulated pig islets and alginate biocompatibility in primates: proof of concept. Transplantation 81: 1345–1353. 16699465 10.1097/01.tp.0000208610.75997.20

[7] Sun Y, Ma X, Zhou D, Vacek I, Sun AM (1996) Normalization of diabetes in spontaneously diabetic cynomologus monkeys by xenografts of microencapsulated porcine islets without immunosuppression. J Clin Invest 98: 1417–1422. 8823307 10.1172/JCI118929 PMC507568

[8] Elliott RB, Escobar L, Calafiore R, Basta G, Garkavenko O, et al (2005) Transplantation of micro- and macroencapsulated piglet islets into mice and monkeys. Transplant Proc 37: 466–469. 15808678 10.1016/j.transproceed.2004.12.198

[9] Gray DW (1997) Encapsulated islet cells: the role of direct and indirect presentation and the relevance to xenotransplantation and autoimmune recurrence. Br Med Bull 53: 777–788. 9536527 10.1093/oxfordjournals.bmb.a011647

[10] Gray DW (2001) An overview of the immune system with specific reference to membrane encapsulation and islet transplantation. Ann N Y Acad Sci 944: 226–239. 11797672 10.1111/j.1749-6632.2001.tb03835.x

[11] Komoda H, Miyagawa S, Kubo T, Kitano E, Kitamura H, et al (2004) A study of the xenoantigenicity of adult pig islets cells. Xenotransplantation 11: 237–246. 15099203 10.1111/j.1399-3089.2004.00121.x

[12] Tam SK, de Haan BJ, Faas MM, Halle JP, Yahia L, et al (2009) Adsorption of human immunoglobulin to implantable alginate-poly-L-lysine microcapsules: effect of microcapsule composition. J Biomed Mater Res A 89: 609–615. 18435412 10.1002/jbm.a.32002

[13] Dufrane D, Goebbels RM, Gianello P (2010) Alginate macroencapsulation of pig islets allows correction of streptozotocin-induced diabetes in primates up to 6 months without immunosuppression. Transplantation 90: 1054–1062. 20975626 10.1097/TP.0b013e3181f6e267

[14] Ludwig B, Zimerman B, Steffen A, Yavriants K, Azarov D, et al (2010) A novel device for islet transplantation providing immune protection and oxygen supply. Horm Metab Res 42: 918–922. 21031332 10.1055/s-0030-1267916

[15] Barkai U, Weir GC, Colton CK, Ludwig B, Bornstein SR, et al.. (2013) Enhanced oxygen supply improves islet viability in a new bioartificial pancreas. Cell Transplant. doi:dx.doi.org/10.3727/096368912X657341. 10.3727/096368912X657341 23043896

[16] Kuzmina A, Hadad U, Fujinaga K, Taube R (2012) Functional characterization of a human cyclin T1 mutant reveals a different binding surface for Tat and HEXIM1. Virology 426: 152–161. 22342181 10.1016/j.virol.2012.01.033

[17] Hyder A, Laue C, Schrezenmeir J (2005) Effect of the immunosuppressive regime of Edmonton protocol on the long-term in vitro insulin secretion from islets of two different species and age categories. Toxicol In Vitro 19: 541–546. 15826812 10.1016/j.tiv.2005.01.005

[18] Lohmann T, List C, Lamesch P, Kohlhaw K, Wenzke M, et al (2000) Diabetes mellitus and islet cell specific autoimmunity as adverse effects of immunsuppressive therapy by FK506/tacrolimus. Exp Clin Endocrinol Diabetes 108: 347–352. 10989953 10.1055/s-2000-8127

[19] Biarnes M, Montolio M, Nacher V, Raurell M, Soler J, et al (2002) Beta-cell death and mass in syngeneically transplanted islets exposed to short- and long-term hyperglycemia. Diabetes 51: 66–72. 11756324 10.2337/diabetes.51.1.66

[20] Shapiro AM, Ricordi C, Hering BJ, Auchincloss H, Lindblad R, et al (2006) International trial of the Edmonton protocol for islet transplantation. N Engl J Med 355: 1318–1330. 17005949 10.1056/NEJMoa061267

[21] Carlsson PO, Palm F, Andersson A, Liss P (2001) Markedly decreased oxygen tension in transplanted rat pancreatic islets irrespective of the implantation site. Diabetes 50: 489–495. 11246867 10.2337/diabetes.50.3.489

[22] Jansson L (1994) The regulation of pancreatic islet blood flow. Diabetes Metab Rev 10: 407–416. 7796706 10.1002/dmr.5610100405

[23] Jansson L, Bodin B, Kallskog O, Andersson A (2005) Duct ligation and pancreatic islet blood flow in rats: physiological growth of islets does not affect islet blood perfusion. Eur J Endocrinol 153: 345–351. 16061842 10.1530/eje.1.01966

[24] Lehmann R, Zuellig RA, Kugelmeier P, Baenninger PB, Moritz W, et al (2007) Superiority of small islets in human islet transplantation. Diabetes 56: 594–603. 17327426 10.2337/db06-0779

[25] McCall M, Shapiro AM (2012) Update on islet transplantation. Cold Spring Harb Perspect Med 2: a007823 doi:10.1101/cshperspect.a007823 22762022 10.1101/cshperspect.a007823 PMC3385934

[26] Dulong JL, Legallais C (2007) A theoretical study of oxygen transfer including cell necrosis for the design of a bioartificial pancreas. Biotechnol Bioeng 96: 990–998. 16897784 10.1002/bit.21140

[27] Storrs R, Dorian R, King SR, Lakey J, Rilo H (2001) Preclinical development of the Islet Sheet. Ann N Y Acad Sci 944: 252–266. 11797674 10.1111/j.1749-6632.2001.tb03837.x

[28] Ludwig B, Ziegler CG, Schally AV, Richter C, Steffen A, et al (2010) Agonist of growth hormone-releasing hormone as a potential effector for survival and proliferation of pancreatic islets. Proc Natl Acad Sci U S A 107: 12623–12628. 20616039 10.1073/pnas.1005098107 PMC2906543

[29] de Groot M, Schuurs TA, Keizer PP, Fekken S, Leuvenink HG, et al (2003) Response of encapsulated rat pancreatic islets to hypoxia. Cell Transplant 12: 867–875. 14763506

[30] Cooper DK, Koren E, Oriol R (1994) Clinical potential of xenotransplantation. Transplant Proc 26: 1331–1332. 7518129

[31] Specke V, Plesker R, Wood J, Coulibaly C, Suling K, et al (2009) No in vivo infection of triple immunosuppressed non-human primates after inoculation with high titers of porcine endogenous retroviruses. Xenotransplantation 16: 34–44. 19243559 10.1111/j.1399-3089.2009.00508.x

